# Association of Tumor Size With Prognosis in Patients With Resectable Endometrial Cancer: A SEER Database Analysis

**DOI:** 10.3389/fonc.2022.887157

**Published:** 2022-06-23

**Authors:** Xuefei Hou, Suru Yue, Jie Liu, Zhiqing Qiu, Liming Xie, Xueying Huang, Shasha Li, Liren Hu, Jiayuan Wu

**Affiliations:** ^1^ Clinical Research Service Center, Affiliated Hospital of Guangdong Medical University, Zhanjiang, China; ^2^ Guangdong Engineering Research Center of Collaborative Innovation Technology of Clinical Medical Big Data Cloud Service in Medical Consortium of West Guangdong Province, Affiliated Hospital of Guangdong Medical University, Zhanjiang, China; ^3^ Department of Pharmacy, Affiliated Hospital of Guangdong Medical University, Zhanjiang, China

**Keywords:** endometrial cancer, tumor size, prognosis, SEER database, death risk

## Abstract

This study aimed to explore the relationship between tumor size (Ts) and prognosis in endometrial cancer (EC). A total of 52,208 patients with EC who underwent total hysterectomy were selected from the Surveillance, Epidemiology, and End Results Program database. Overall survival (OS) and endometrial cancer-specific survival (ESS) were chosen as survival outcomes. The Cox proportional hazards model was used to explore the effect of Ts on prognosis. The restricted cubic splines based on the Cox regression model were used to determine the nonlinear relationship between Ts and survival. When Ts was analyzed as a categorical variable, the risk of death increased with Ts, with the highest risk in patients with Ts > 9 cm with regard to all-cause death (ACD) (hazard ratio [HR] 1.317; 95% confidence interval [CI], 1.196-1.450; *P* < 0.001) and endometrial cancer-specific death (ESD) (HR, 1.378; 95% CI, 1.226-1.549; *P* < 0.001). As a continuous variable, Ts showed a nonlinear relationship with ACD (HR, 1.061; 95% CI, 1.053-1.069; *P* < 0.001) and ESD (HR, 1.062; 95% CI, 1.052-1.073; *P* < 0.001). The risk of mortality increased quickly with Ts when Ts was less than 7.5 cm and then leveled off when Ts was larger than 7.5 cm in all patients. Among patients with lymph node metastasis, the risk of poor prognosis decreased rapidly with Ts when Ts was less than 3.5 cm, and subsequently increased sharply with Ts when Ts ranged from 3.5 cm to 7.5 cm, and then increased slowly when Ts was larger than 7.5 cm (*P* < 0.001 for nonlinearity). There was a nonlinear relationship between Ts and prognosis in patients with EC. Clinicians should not ignore the impact of small tumors on prognosis in EC patients with lymph node metastasis.

## Introduction

According to the latest statistics from the Global Cancer Observatory, endometrial cancer (EC) was ranked third in gynecological tumors, with an estimated 417,367 new cases and 97,370 deaths around the world in 2020 with an increase of 9.2% and 8.3%, respectively, compared to those in 2018. (1, 2) Reducing the recurrence rate and prolonging survival time were the goals for clinician to improve the prognosis of patients with EC, as current medical methods cannot completely cure this disease. (3–5)

Currently, the American Joint Committee on Cancer (AJCC) and the International Federation of Gynecology and Obstetrics (FIGO) staging system have been widely used for prognostic prediction and treatment selection in patients with EC. However, the prognosis of patients with the same stage varies dramatically; thus, management according to the tumor staging system may lead to undertreatment, as Marcos et al. (6) found that 10% of women with low-risk EC (type 1, stage IA grade 1 or 2) and 15% of women with intermediate-risk EC (type 1, stage IA grade 3, or stage IB grade 1 or 2) suffer from lymph node metastasis (LNM) according to FIGO staging system. Therefore, additional tools are needed to improve the management of patients with EC to accommodate surgical staging and adjuvant therapy.

Tumor size (Ts) was first reported as a prognostic indicator of EC in the 1980s. (3) Since then, many investigators have examined the prognostic significance of Ts. Thus far, studies have observed that Ts is an independent predictive factor for LNM, recurrence, and prognosis of EC. (4, 7–9) Mariam et al. (10) revealed that the combination of preoperative biopsy and intraoperative Ts could improve the accuracy of surgical staging. They suggested that among patients with preoperative histological grade 1 or 2, lymphadenectomy was recommended for those with Ts > 2 cm if an accurate frozen section was lacking, but not for those with Ts ≤ 2 cm. Although evidence has shown that Ts can be used as a prognostic indicator in EC, it has not yet been included in the tumor-nodes-metastasis staging system, possibly because the relationship between Ts and prognosis of EC is still controversial. Ozgul et al. (11) conducted a retrospective study based on 250 patients with stage II EC and found that Ts was not associated with five-year disease-free survival and overall survival (OS). Moreover, Shah et al. (12) had the same results in a study involving 345 surgically treated EC patients. Doll et al. (13) observed no association between Ts > 2 cm and recurrence in high-grade EC.

To date, studies on the association between Ts and the prognosis of EC have mainly focused on the survival differences among different Ts categories. (14–16) However, this method cannot reflect the effect of Ts on prognosis in detail. Some evidence has shown that the relationship between Ts and prognosis is nonlinear in a variety of cancers. (17) Based on the available evidence, we hypothesized that Ts and prognosis of EC may have a complex rather than a simple linear relationship, and the effects of different Ts on the risk of mortality might be distinct in these patients. Therefore, this study aims to better characterize the relationship between Ts and prognosis based on a large sample of EC patients who underwent surgery and to provide evidence for revising the tumor staging system.

## Materials and Methods

### Study Population

The data for the study were extracted from the Surveillance, Epidemiology, and End Results Program (SEER) database by using the SEER*Stat software (version 8.3.9.2, National Cancer Institute, Bethesda, MD), the cases we chosen were registered in SEER between 2004 and 2018. The SEER database covers 28% of the US population from 18 cancer registries and is one of the largest population-based cancer registries in the world. Institutional ethical approval and informed consent are not required for this study because the SEER database is anonymous and freely available to the public.

In the study, we utilized the Incidence-SEER Research Data, 18 Registries, Nov 2020 Sub (2000–2018) registry as the data source. All patients diagnosed with EC (site recode ICD-O-3/WHO 2008 of corpus uteri, behavior recode ICD-O-3 of “malignant,” histology type ICD-O-3 of “8140-8389 and 8440-8499”) who underwent surgery were included in this study. Inclusion criteria for patients were as follows: (1) diagnosis with EC as the first and only cancer; (2) age at diagnosis ≥ 18 years; (3) patients underwent total hysterectomy; (4) patients had complete postoperative follow-up data.

### Variable Selection

**Figure 1:**
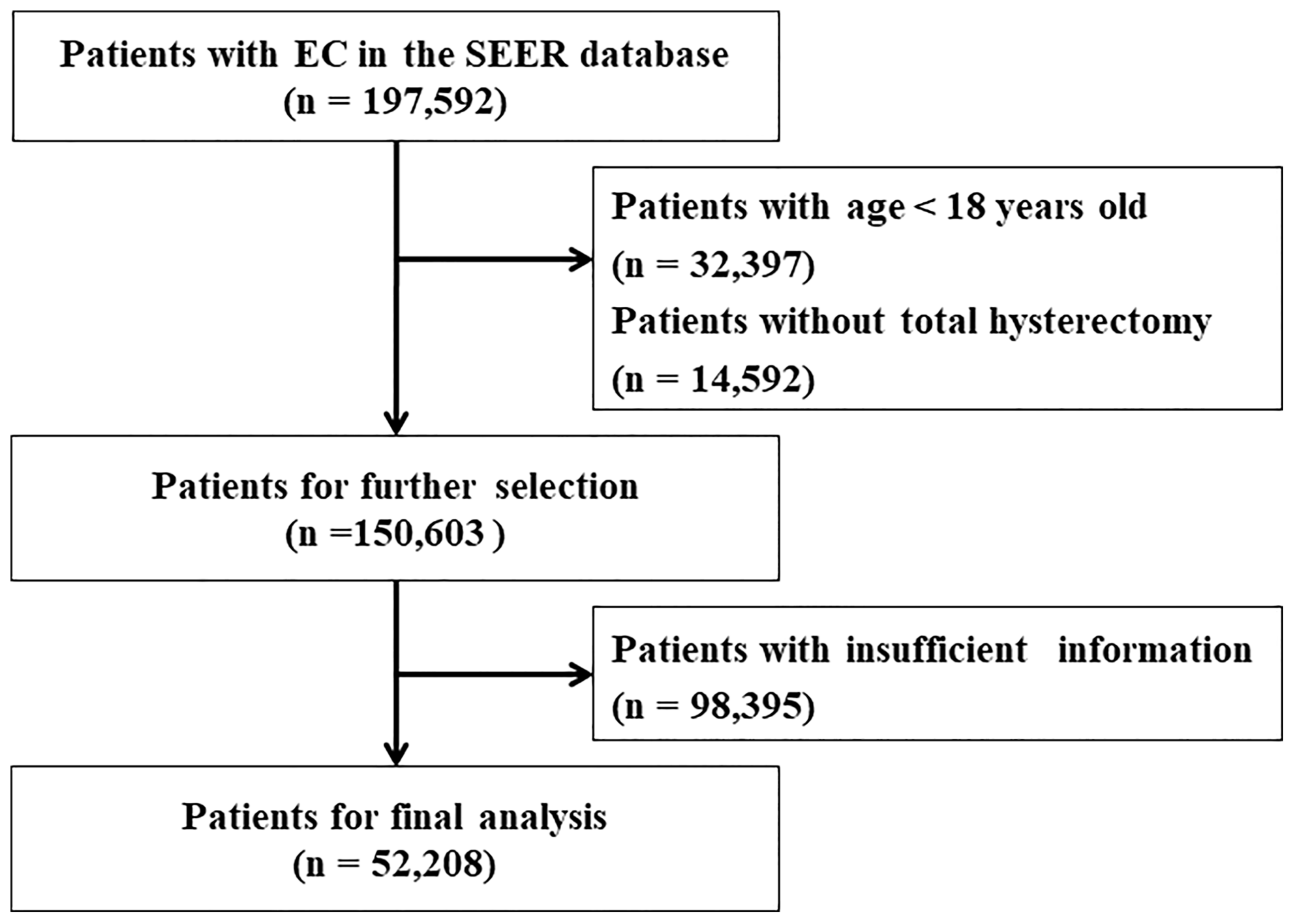
Flow chart for screening eligible patients. EC, endometrial cancer. SEER, the Surveillance, Epidemiology, and End Results Program database.

Information including age, race, histological type, grade, stage, Ts, number of nodes examined, lymph node (LN) status, follow-up time and tumor number were extracted from the SEER database. Age was divided into four groups (18–56, 57–61, 62–69, and 70+ years) according to the X-tile software. Race was classified as white, black, and others. The histological type included endometrial endometrioid adenocarcinoma (EEA, codes: 8140–8389) and serous endometrioid adenocarcinoma (SEA, codes: 8440–8499) by using the ICD-O-3 codes. The eighth edition of the AJCC staging system was applied to patients in this study. Data recorded using the sixth and seventh editions were converted to the eighth edition system. The tumor grades were grouped as Grade I (well-differentiated), Grade II (moderately differentiated), Grade III (poorly differentiated), and Grade IV (undifferentiated or anaplastic), and the TNM stages consisted of stage I to stage IV. Ts was divided into 10 subgroups: Group 1 (≤1 cm), Group 2 (1.1−2 cm), Group 3 (2.1−3 cm), Group 4 (3.1−4 cm), Group 5 (4.1−5 cm), Group 6 (5.1−6 cm), Group 7 (6.1−7 cm), Group 8 (7.1−8 cm), Group 9 (8.1−9 cm), and Group 10 (> 9 cm). Overall survival (OS) and endometrial cancer-specific survival (ESS) were chosen as survival outcomes. OS was defined as the period from diagnosis until death from any cause, and ESS was defined as the period from diagnosis until death from EC. The process of variable selection was showed in **Figure 1**.

### Statistical Analysis

The distribution of the variables was evaluated with the Shapiro-Wilk test. Normally distributed variables were expressed as mean and standard deviation (SD), non-normally distributed variables were reported as medians with interquartile ranges (IQRs) and compared using the Wilcoxon rank-sum test, while categorical variables were expressed as number and percentage and compared using the chi-squared test. The time-dependent ROC curve was used to calculate optimal cut-offs of tumor size according to final survival status. The Kaplan-Meier method was used to calculate OS and ESS. The univariate and multivariate Cox proportional hazards models were used to estimate hazard ratios (HR) and 95% confidence intervals (CI). The restricted cubic spline analysis (RCS) for the Cox model was used to flexibly model and visualize the association between Ts and prognosis (18). Subgroup analyses for patients with and without LNM were conducted to further examine the effects of Ts on survival outcomes. All statistical analyses were performed using *R* software version 4.0.5, and a two-sided *P* value < 0.05 was considered statistically significant.

## Results

### Characteristics of Patients

**Table 1** shows the details of the patient characteristics. A total of 52,208 patients were involved in this study, with an average age of 62.9 ± 10.7 years and a median Ts of 3.5 cm. The number of nodes examined and the follow-up median times were 13 (6−21) and 56 (23−100) months, respectively. There were 13,715 (26.3%), 9719 (18.6%), 14,891 (28.5%), and 13,888 (26.6%) patients in the age groups of 18−56, 57−61, 62−59, and 70+ years, respectively. Most patients were white (n = 42,265, 81.0%), had a histological type of EEA (n = 47,127, 90.3%), with stage I cancer (n = 36,108, 69.2%). The numbers of patients with grade 1, grade 2, grade 3, and grade 4 tumors were 18,780 (36.0%), 17,047 (32.7%), 12,556 (24.0%), and 3825 (7.3%), respectively. More than half of the patients were LN negative (n = 44,982, 86.2%) and had one tumor (n = 41,342, 79.2%).

**Table 1:** Baseline characteristics of patients with endometrial cancer according to tumor size categories.

Variable	Overall	Ts categories (cm)									
		≤ 1	1.1−2	2.1−3	3.1−4	4.1−5	5.1−6	6.1−7	7.1−8	8.1−9	> 9
Age (years), n (%)											
18−56	13715 (26.3)	1158 (8.4)	1822 (13.3)	2304 (16.8)	2434 (17.7)	1915 (14.0)	1359 (9.9)	908 (6.6)	658 (4.8)	360 (2.6)	797 (5.8)
57−61	9719 (18.6)	703 (7.2)	1405 (14.5)	2010 (20.7)	1865 (19.2)	1419 (14.6)	860 (8.8)	547 (5.6)	330 (3.4)	202 (2.1)	378 (3.9)
62−69	14891 (28.5)	1095 (7.4)	2087 (14.0)	3066 (20.6)	3055 (20.5)	2257 (15.2)	1344 (9.0)	727 (4.9)	467 (3.1)	279 (1.9)	514 (3.5)
70+	13883 (26.6)	771 (5.6)	1856 (13.4)	2811 (20.2)	2995 (21.6)	2262 (16.3)	1307 (9.4)	749 (5.4)	444 (3.2)	264 (1.9)	424 (3.1)
Histological type, n (%)											
EEA	47127 (90.3)	3256 (6.9)	9281 (19.7)	9475 (20.1)	7121 (15.1)	4389 (9.3)	2644 (5.6)	1696 (3.6)	966 (2.0)	1866 (4.0)	6433 (13.7)
SEA	5081 (9.7)	471 (9.3)	910 (14.5)	874 (17.9)	732 (17.2)	481 (14.4)	287 (9.5)	203 (5.6)	139 (4.0)	247 (2.7)	737 (4.9)
Race, n (%)											
White	42265 (81.0)	3045 (7.2)	5896 (14.0)	8531 (20.2)	8554 (20.2)	6437 (15.2)	3852 (9.1)	2208 (5.2)	1405 (3.3)	825 (2.0)	1512 (3.6)
Black	4452 (8.5)	298 (6.7)	476 (10.7)	614 (13.8)	762 (17.1)	675 (15.2)	522 (11.7)	374 (8.4)	270 (6.1)	142 (3.2)	319 (7.2)
Others	5491 (10.5)	384 (7.0)	798 (14.5)	1046 (19.0)	1033 (18.8)	741 (13.5)	496 (9.0)	349 (6.4)	224 (4.1)	138 (2.5)	282 (5.1)
Grade, n (%)											
G1	18780 (36.0)	1849 (9.8)	3188 (17.0)	4011 (21.4)	3764 (20.0)	2559 (13.6)	1426 (7.6)	764 (4.1)	486 (2.6)	278 (1.5)	455 (2.4)
G2	17047 (32.7)	937 (5.5)	2145 (12.6)	3461 (20.3)	3587 (21.0)	2699 (15.8)	1683 (9.9)	987 (5.8)	604 (3.5)	323 (1.9)	621 (3.6)
G3	12556 (24.0)	661 (5.3)	1412 (11.2)	2076 (16.5)	2324 (18.5)	1998 (15.9)	1366 (10.9)	925 (7.4)	633 (5.0)	381 (3.0)	780 (6.2)
G4	3825 (7.3)	280 (7.3)	425 (11.1)	643 (16.8)	674 (17.6)	597 (15.6)	395 (10.3)	255 (6.7)	176 (4.6)	123 (3.2)	257 (6.7)
Stage, n (%)											
I	36108 (69.2)	3243 (9.0)	5938 (16.4)	8026 (22.2)	7549 (20.9)	5123 (14.2)	2845 (7.9)	1494 (4.1)	837 (2.3)	425 (1.2)	628 (1.7)
II	4356 (8.3)	157 (3.6)	406 (9.3)	675 (15.5)	815 (18.7)	714 (16.4)	515 (11.8)	356 (8.2)	259 (5.9)	167 (3.8)	292 (6.7)
III	9861 (18.9)	276 (2.8)	712 (7.2)	1294 (13.1)	1728 (17.5)	1755 (17.8)	1265 (12.8)	893 (9.1)	640 (6.5)	392 (4.0)	906 (9.2)
IV	1883 (3.6)	51 (2.7)	114 (6.1)	196 (10.4)	257 (13.6)	261 (13.9)	245 (13.0)	188 (10.0)	163 (8.7)	121 (6.4)	287 (15.2)
Lymph node status, n (%)											
Negative	44982 (86.2)	3567 (7.9)	6760 (15.0)	9397 (20.9)	9166 (20.4)	6597 (14.7)	3903 (8.7)	2198 (4.9)	1365 (3.0)	754 (1.7)	1275 (2.8)
Positive	7226 (13.8)	1600 (2.2)	410 (5.7)	794 (11.0)	1183 (16.4)	1256 (17.4)	967 (13.4)	733 (10.1)	534 (7.4)	351 (4.9)	838 (11.6)
Tumor number, n (%)											
Single	41342 (79.2)	2879 (7.0)	5514 (13.3)	7997 (19.3)	8210 (19.9)	6231 (15.1)	3892 (9.4)	2388 (5.8)	1561 (3.8)	909 (2.2)	1761 (4.3)
Multiple	10866 (20.8)	848 (7.8)	1656 (15.2)	2194 (20.2)	2139 (19.7)	1622 (14.9)	978 (9.0)	543 (5.0)	338 (3.1)	196 (1.8)	352 (3.2)
Number of nodes examined [Median (IQR)]	13 (6-21)										

SD, standard deviation; IQR, interquartile range; EEA, endometrial endometrioid adenocarcinoma; SEA, serous endometrioid adenocarcinoma.

### Association Between Ts and Prognosis

The optimal cut-offs of tumor size were 3.9 cm in OS and 4.0 cm in ESS, which was calculated by the time-dependent ROC curve (**Supplementary Figure 1**). So, we defined group (3.1-4 cm) as a reference when tumor size was analyzed as a categorical variable.

When Ts was analyzed as a categorical variable, the univariate Cox regression models showed that the risk of all-cause death (ACD) and endometrial cancer-specific death (ESD) gradually increased as the tumor grew (**Table 2**, Model 1 and Model 2). As compared with patients with Ts of 3.1−4 cm (the reference group), the highest risk of ACD and ESD was observed in patients with Ts > 9 cm with HRs of 2.29 (95% CI, 2.10−2.49; *P* < 0.001) and 3.17 (95% CI, 2.87−3.51; *P* < 0.001), respectively, whereas the lowest risk was observed in patients with Ts ≤ 1cm with HRs of 0.56 (95% CI, 0.51−0.62; *P* < 0.001) and 0.52 (95% CI, 0.45−0.60; *P* < 0.001), respectively. After adjustment for confounding factors of which *P* < 0.05 in univariate analysis, the multivariate Cox regression analysis indicated that patients with large Ts were prone to suffer a high risk of death, with the highest HRs of 1.61 (95% CI, 1.48−1.76; *P)* for ACD and 1.61 (95% CI, 1.46−1.79; *P* < 0.05) for ESD in patients with Ts > 9 cm, compared with patients with Ts of 3.1−4 cm (the reference group) (**Table 2**, Model 3 and Model 4). The results of multivariate analyses for Ts as categorical variables in all patients are listed in **Supplementary Table 1**.

**Table 2:** Univariate and multivariate Cox regression analyses of ACD and CSD according to Ts in patients with endometrial cancer.

Ts	HR (95% CI)							
	Model 1 (ACD)	*P*	Model 2 (ESD)	*P*	Model 3 (ACD)	*P*	Model 4 (ESD)	*P*
≤ 1 cm	0.561 (0.506−0.622)	< 0.001	0.518 (0.448−0.599)	< 0.001	0.731 (0.659−0.811)	< 0.001	0.744 (0.642−0.861)	< 0.001
1.1−2 cm	0.709 (0.658−0.763)	< 0.001	0.650 (0.585−0.721)	< 0.001	0.826 (0.767−0.891)	0.024	0.829 (0.747−0.921)	0.024
2.1−3 cm	0.806 (0.755−0.860)	< 0.001	0.745 (0.680−0.815)	< 0.001	0.880 (0.824−0.939)	< 0.001	0.864 (0.789−0.946)	< 0.001
3.1−4 cm	1 (reference)		1 (reference)		1 (reference)		1 (reference)	
4.1−5 cm	1.274 (1.196−1.358)	< 0.001	1.359 (1.249−1.479)	< 0.001	1.151 (1.080−1.227)	< 0.001	1.170 (1.075−1.273)	< 0.001
5.1−6 cm	1.442 (1.343−1.549)	< 0.001	1.622 (1.479−1.779)	< 0.001	1.230 (1.145−1.321)	< 0.001	1.232 (1.123−1.352)	< 0.001
6.1−7 cm	1.637 (1.510−1.776)	< 0.001	2.051 (1.854−2.268)	< 0.001	1.335 (1.230−1.449)	< 0.001	1.406 (1.270−1.557)	< 0.001
7.1−8 cm	1.669 (1.517−1.836)	< 0.001	2.171 (1.934−2.438)	< 0.001	1.317 (1.196−1.450)	< 0.001	1.378 (1.226−1.549)	< 0.001
8.1−9 cm	1.908 (1.702−2.139)	< 0.001	2.617 (2.289−2.991)	< 0.001	1.416 (1.262−1.589)	< 0.001	1.514 (1.323−1.733)	< 0.001
> 9 cm	2.291 (2.104−2.494)	< 0.001	3.169 (2.865−3.506)	< 0.001	1.613 (1.478−1.760)	< 0.001	1.614 (1.455−1.790)	< 0.001
Ts+	1.092 (1.086−1.099)	< 0.001	1.101 (1.095−1.108)	< 0.001	1.061 (1.053−1.069)	< 0.001	1.062 (1.052−1.073)	< 0.001

Model 1: Results of univariate Cox proportional hazards models for ACD. Model 2: Results of univariate Cox proportional hazards models for ESD. Model 3: Results of multivariate Cox proportional hazards models for ACD after adjustment for age, histological type, race, grade, stage, lymph node status, number of lymph node examined, and tumor number. Mode 4: Results of multivariate Cox proportional hazards models for ESD after adjustment for age, histological type, race, grade, stage, lymph node status, number of lymph node examined, and tumor number. Ts+: Ts was analyzed as a continuous variable. Ts, tumor size; HR, hazard ratio; CI, confidence interval; ACD, all-cause death; ESD, endometrial cancer-specific death.

**Figure 2:**
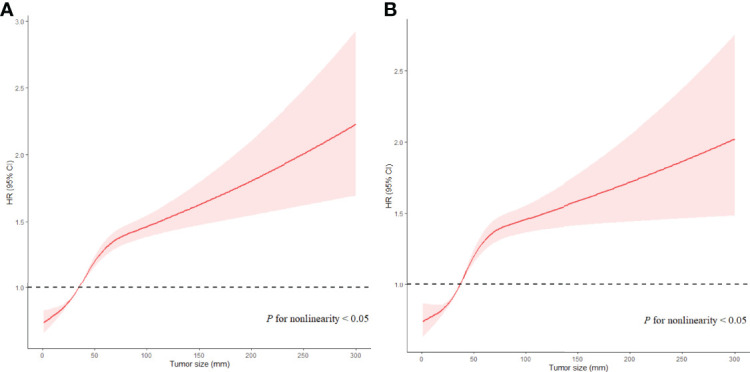
Associations of Ts with prognosis in EC patients in Cox models with RCS after adjustment. Red lines estimated HR of Ts; shadow area 95% CI. **(A)** Adjusted RCS model for ACD. **(B)** Adjusted RCS model for ESD.

When Ts was analyzed as a continuous variable, an increased Ts was also significantly associated with a high risk of ACD (HR, 1.092; 95% CI, 1.049–1.066; *P* < 0.001) and ESD (HR, 1.101; 95% CI, 1.095–1.108; *P* < 0.001). In the fully adjusted model (**Table 2**, Model 3 and Model 4), a larger Ts also indicated a higher risk of ACD (HR, 1.06; 95% CI, 1.05−1.07; *P* < 0.001) and ESD(HR, 1.06; 95% CI, 1.05−1.07; *P* < 0.001). In the RCS model, there is a nonlinear relationship between Ts and prognosis (*P* < 0.001 for nonlinearity), with a trend toward rising rapidly and then gradually (**Figure 2**). Taking the value of 7.5 cm as a turning point, the slope of the low Ts part (< 7.5 cm) was steeper than that of the high part (≥ 7.5 cm). The results of multivariate analyses for Ts as continuous variables in all patients are listed in **Supplementary Table 2**.

### Subgroup Analyses

**Figure 3:**
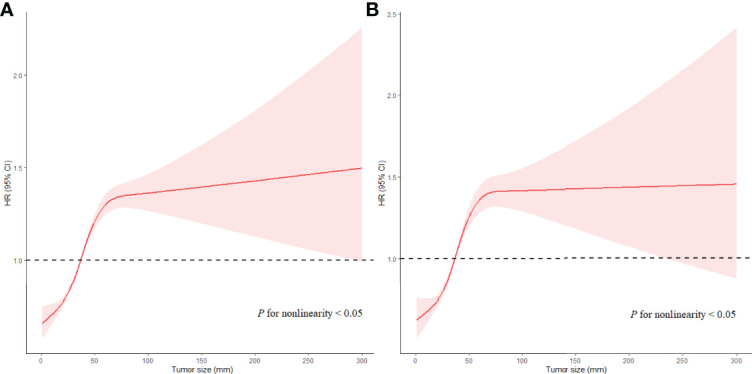
Associations of Ts with prognosis in EC patients with LNM in RCS with Cox models after adjustment. Red lines estimated hazard ratio of tumor size; shadow area 95% CI. **(A)** Adjusted RCS model for ACD. **(B)** Adjusted RCS model for ESD.

To further explore the relationship between Ts and prognosis in different LN statuses, all patients were divided into two groups, namely, LNM (N=7,226) and non-LNM (N=44,982). For patients without LNM, the fully-adjusted Cox regression models showed that the highest risk of ACD (HR, 1.457; 95% CI, 1.284−1.653; *P* < 0.001) and ESD (HR, 1.702; 95% CI, 1.471−1.970; *P* < 0.001) was observed in patients with Ts > 9 cm as compared to the risk in patients with Ts of 3.1−4 cm (the reference group), when Ts was analyzed as a categorical variable (**Table 3**). The results of multivariate analyses for Ts as categorical variables in patients without LNM are listed in **Supplementary Tables 3**. When Ts was analyzed as a continuous variable, Ts was independently associated with ACD (HR, 1.067; 95% CI, 1.057−1.077; *P* < 0.001) and ESD (HR, 1.075; 95% CI, 1.061−1.088; *P* < 0.001) in the fully adjusted models (**Table 3**). Ts also showed a nonlinear relationship with OS (*P* < 0.001 for nonlinearity) and ESS (*P* < 0.001 for nonlinearity) (**Figure 3**). The results of multivariate analyses for Ts as continuous variables in patients without LNM are listed in **Supplementary Tables 4**.

**Table 3:** Multivariate Cox regression analyses of ACD and ESD according to Ts in patients with endometrial cancer according to LNM.

Ts	HR (95% CI)							
	Without LNM				With LNM			
	Model 1 (ACD)	*P*	Model 2 (ESD)	*P*	Model 1 (ACD)	*P*	Model 2 (ESD)	*P*
≤ 1 cm	0.658 (0.587−0.738)	< 0.001	0.732 (0.646−0.829)	< 0.001	1.254 (0.982−1.601)	0.336	1.156 (0.953−1.401)	0.272
1.1−2 cm	0.771 (0.710−0.838)	0.009	0.811 (0.727−0.905)	0.066	1.097 (0.922−1.306)	0.087	0.978 (0.830−1.153)	0.055
2.1−3 cm	0.840 (0.781−0.904)	< 0.001	1.211 (1.090−1.347)	0.002	1.004 (0.869−1.161)	0.070	1.108 (0.962−1.275)	0.066
3.1−4 cm	1 (reference)		1 (reference)		1 (reference)		1 (reference)	
4.1−5 cm	1.175 (1.090−1.266)	< 0.001	1.267 (1.123−1.429)	< 0.001	1.112 (0.981−1.259)	0.331	1.195 (1.031−1.385)	0.593
5.1−6 cm	1.262 (1.159−1.375)	< 0.001	1.468 (1.278−1.685)	< 0.001	1.175 (1.029−1.341)	0.604	1.360 (1.166−1.586)	0.687
6.1−7 cm	1.382 (1.246−1.533)	< 0.001	1.491 (1.273−1.747)	< 0.001	1.320 (1.149−1.516)	0.687	1.304 (1.094−1.554)	0.924
7.1−8 cm	1.350 (1.192−1.528)	< 0.001	1.680 (1.386−2.037)	< 0.001	1.279 (1.092−1.499)	0.879	1.395 (1.150−1.693)	0.588
8.1−9 cm	1.454 (1.245−1.698)	< 0.001	1.443 (1.228−1.696)	< 0.001	1.359 (1.138−1.624)	0.562	1.702 (1.471−1.970)	0.039
9.1−10 cm	1.457 (1.284−1.653)	< 0.001	1.702 (1.471−1.970)	< 0.001	1.286 (0.983−1.681)	0.030	1.156 (0.953−1.401)	< 0.001
Ts+	1.067 (1.057-1.077)	< 0.001	1.075 (1.061-1.088)	< 0.001	1.047 (1.032-1.062)	< 0.001	1.047 (1.032-1.063)	< 0.001

Model 1: Results of multivariate Cox proportional hazards models for ACD after adjustment for age, histological type, race, grade, stage, lymph node status, number of lymph node examined, and tumor number. Mode 2: Results of multivariate Cox proportional hazards models for ESD after adjustment for age, histological type, race, grade, stage, lymph node status, number of lymph node examined, and tumor number. Ts+: Ts was analyzed as a continuous variable. Ts, tumor size; HR, hazard ratio; CI, confidence interval; ACD, all-cause death; ESD, endometrial cancer-specific death; LNM, lymph node metastasis.

**Figure 4:**
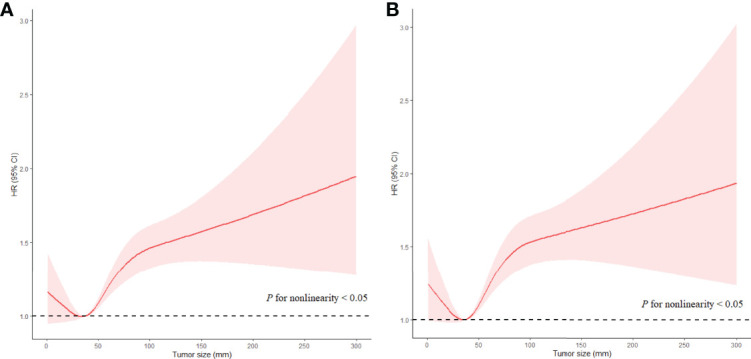
Associations of Ts with prognosis in EC patients without LNM in RCS with Cox models after adjustment. Red lines estimated HR of Ts; shadow area 95%CI. **(A)** Adjusted RCS model for ACD. **(B)** Adjusted RCS model for ESD.

For patients with LNM, the highest HR of Ts was 1.359 (95% CI, 1.138−1.624; *P* < 0.05) for ACD and 1.702 (95% CI, 1.471−1.970; *P* < 0.05) for ESD in patients with Ts > 9 cm as compared with those in patients with Ts of 3.1−4 cm (the reference group) when Ts was analyzed as a categorical variable. The results of multivariate analyses for Ts as categorical variables in patients with LNM are listed in **Supplementary Tables 5**. When Ts was analyzed as a continuous variable, Ts was independently associated with ACD (HR, 1.047; 95% CI, 1.032−1.062; *P* < 0.05) and ESD (HR, 1.047; 95% CI, 1.032−1.063; *P* < 0.05) in the fully adjusted models (**Table 3**). A nonlinear relationship was also found between Ts and prognosis of EC (*P* < 0.05 for nonlinearity), with the risk of poor prognosis decreasing quickly with Ts when Ts was less than 3.5 cm, subsequently increasing rapidly with Ts when Ts ranged from 3.5 cm to 7.5 cm, and then increasing slowly when Ts was larger than 7.5 cm (*P* < 0.05 for nonlinearity) (**Figure 4**). The results of multivariate analyses for Ts as continuous variables in patients with LNM are listed in **Supplementary Tables 6**.

## Discussion

Our results indicated that large Ts was significantly associated with poor survival outcomes in patients with resectable EC. Among all patients with EC, we observed a nonlinear relationship between Ts and prognosis (*P* < 0.05 for nonlinearity), with a trend toward rising rapidly and then gradually. Among patients with LNM, the risk of poor prognosis decreased quickly with Ts when Ts was less than 3.5 cm, subsequently increasing rapidly with Ts when Ts ranged from 3.5 cm to 7.5 cm, and then increasing slowly when Ts was larger than 7.5 cm.

The T staging of EC in AJCC and FIGO systems is classified by the degree of tumor invasion and whether it is confined to the uterus. Ts has not been adopted in the staging system in EC but has been used in other cancers, such as cervical cancer, liver cancer, and pancreatic cancer. (19, 20) In cervical cancer, patients with the deepest invasion of ≥ 5 mm and lesion limited to the cervix uteri were grouped as stage IB. In the more detailed division, patients with a depth of stromal invasion ≥ 5 cm and Ts < 2 cm can be classified as IB1, patients with Ts of 2 to 4 cm can be grouped into IB2, and patients with Ts ≥ 4 cm can be categorized as IB3. (21) Similarly, Ts has also been used in the staging system of vaginal cancer. The patients with vaginal cancer only in the vagina were grouped into two stages: T1a (Ts ≤ 2.0 cm) and T1b (Ts > 2.0 cm). Patients with vaginal cancer whose tumor grew through the vaginal wall but did not reach the pelvic wall were divided into two stages: T2a (Ts ≤ 2.0 cm) and T2b (Ts >2.0 cm). (22) In the previous study, some scholars had proposed adopting Ts in the staging system of EC. Roberto et al. (23) suggested that Ts should be a useful marker for the surgical staging of EC. Therefore, incorporating Ts into the classification of EC may help to improve the accuracy of tumor staging and provide a basis for doctors to select a better treatment.

In the entire cohort, we observed that the risk of mortality gradually rose as the tumor grew, and larger Ts indicated poorer prognosis in patients with EC. Similarly, Julian et al. (9) demonstrated that the five-year survival rate progressively decreased when the tumor volume grew. As Maraelys et al (24). used three mathematical models (Gompertz, Logistic and Kolmogorov-Johnson-Mehl-Avrami) to imitate unperturbed fibrosarcoma Sa-37 tumor growth, and those models showed the same results that tumor exhibits a sigmoidal kinetics characteristic. Moreover, Laird et al (25). analyzed 19 examples of 12 different tumors in mice, rats, and rabbits and concluded that the growth of a transplanted, or primary, tumor can be well described by the Gompertz equation, that is, the tumor grows at an exponential rate in the early stage, but with the increase of Ts, the growth rate slows down and leveled off. According to the results of RCS, the risk of mortality increased rapidly with the expansion of the Ts (≤ 7.5 cm) and then increased slowly (Ts > 7.5 cm). So, we hypothesized that the tumor cells proliferate rapidly at this stage (Ts ≤ 7.5 cm), and as the Ts increases, the tumor progresses more aggressively, leading to a rapid increase in the risk of mortality. After Ts increases to a certain extent, the tumor proliferation slows down due to the influence of external environmental factors, such as the formation of microenvironments and microvessels, resulting in a slower rate of tumor progression and a slow increase in the risk of death as the curve showed in Ts > 7.5 cm. Moreover, if the tumor is smaller than 7.5 cm, the drug of treatment may choose tumor growth blockers, and if the tumor is larger than 7.5 cm, surgical resection may be better. Based on this study, we only explored the relationship between tumor size and the risk of death, the process of tumor growth is complex and the biological mechanism is not entirely clear, further research is needed on whether the above-mentioned treatment options are feasible.

The effect of Ts on prognosis was significantly different in patients with LNM and those without LNM. It was acknowledged that large Ts is associated with lymph node involvement and poor survival outcomes. (7, 14, 26) However, the risk of mortality decreased rapidly with Ts when the Ts was less than 3.5 cm, indicating that smaller Ts predicted a worse prognosis within this range of Ts in EC patients with LNM. Until now, few studies have examined the effects of small tumors on poor survival in EC. However, some evidence could be found for other cancers. Muralidhar et al (27). observed that patients with small Ts (< 0.1 cm) in prostate cancer suffered from a poorer long-term prognosis than did patients with larger Ts, and small Ts might be associated with LN involvement. Similarly, Wo et al (28). demonstrated that patients with Ts less than 0.5 cm in breast cancer had a lower survival rate compared with patients with Ts larger than 0.5 cm. These studies may support our hypothesis that smaller tumors in EC patients with LNM may represent greater biological aggressiveness and earlier acquisition of genetic changes that promote tumor cell spread to regional or distant sites. As vinayak et al. (27) had same view, they found patients who had LNM in very small prostate cancers presented a particularly aggressive disease variant compared with larger tumors. These small tumors may represent higher mutation rates and thus evade the body’s immune surveillance and anti-tumor immune response. Haffner et al (29). used whole-genome sequencing and molecular to analyze and trace the lineage of cell clones from node-positive patients who eventually died of prostate cancer. They found that lethal clones tended to arise from small tumor and low-grade disease rather than from larger and higher-grade diseases. The reason why these small tumors are more migratory may be that the deregulation of miRNAs, likes miR-142 targetes CCND1 to activate cyclin-dependent kinase (CDK)4/6 for stimulating proliferation, migration, and invasion of cells (30). Mahecha et al. (31) observed that the overexpressed gene of vascular endothelial growth can lead to an increase of the number of new blood vessels in tumor tissues, and the newer blood vessels, the deeper the tumor invasion into myometrium, resulting in vascular metastasis, poor grade, and poor prognosis. Ray et al. (32) found that the overexpression of pro-inflammatory adipocytokines, such as leptin, can also promote the transformation of epithelial mesenchymal to stimulate endometrial cancer growth, proliferation, invasion, and metastasis. Moreover, a larger Ts usually leads to more aggressive treatment, such as a more complete lymphadenectomy and surgical evaluation, resulting in a better prognosis. Therefore, further research on the biological basis of small tumors associated with LNM in EC may discover novel genomic changes, new drug targets, or prognostic markers, thus providing new approaches to guide the selection of treatment options and improve prognosis.

The study also had several limitations. First, due to its retrospective nature, selection bias was inevitable, as the variable of treatment history (radiation, chemotherapy, hormonal therapy) not been included, so the results should be interpreted with caution. Second, in this study, we only selected the variables (EEA, SEA) with large sample sizes, but other pathological types also are worthy of study. And because of the limited classification of races in the SEER database, we could not get detailed information about it. Third, the lack of standardization in pathological classification may result in some patients being misclassified. Fourth, we only extracted prognostic information on OS and ESS, as more information such as recurrence and metastasis cannot be obtained from the database. Finally, the factors affecting tumor growth were complex, but we are unable to simulate the real environment of tumor growth, so there may be a certain gap between the model and the real situation of diagnosis and treatment.

## Conclusion

In this study, we revealed a nonlinear relationship between Ts and prognosis in patients with EC, and the risk of mortality increased monotonically with increasing Ts. However, the effect pattern of Ts on prognosis in patients with LNM was significantly different from that in patients without LNM. Among patients with LNM, a smaller Ts indicated a worse survival outcome when Ts was less than 3.5 cm, suggesting that clinicians should not ignore the impact of small tumor size on prognosis in these patients.

## Data Availability Statement

The original contributions presented in the study are included in the article/**Supplementary Material**. Further inquiries can be directed to the corresponding authors.

## Ethics Statement

Ethical review and approval were not required for the study on human participants in accordance with the local legislation and institutional requirements. Written informed consent for participation was not required for this study in accordance with the national legislation and the institutional requirements.

## Author Contributions

XFH and SY wrote the draft and revised it. JW and LH designed and supervised the study. JL, ZQ, and LX extracted and cleaned the data. XYH and SL designed the figures and tables. All authors contributed to the article and approved the submitted version.

## Conflict of Interest

The authors declare that the research was conducted in the absence of any commercial or financial relationships that could be construed as a potential conflict of interest

## Publisher’s Note

All claims expressed in this article are solely those of the authors and do not necessarily represent those of their affiliated organizations, or those of the publisher, the editors and the reviewers. Any product that may be evaluated in this article, or claim that may be made by its manufacturer, is not guaranteed or endorsed by the publisher.

## Supplementary Material

Supplementary Figure 1 The ROC curve in all EC patients. **(A)** The ROC curve for ACD. **(B)** The ROC curve for ESD.
