# Supplementary figures and images for: Association of Tumor Size With Prognosis in Patients With Resectable Endometrial Cancer: A SEER Database Analysis
Source: Front Oncol. 2022 Jun 23;12:887157. doi: 10.3389/fonc.2022.887157 (PMC9259839; doi:10.3389/fonc.2022.887157)

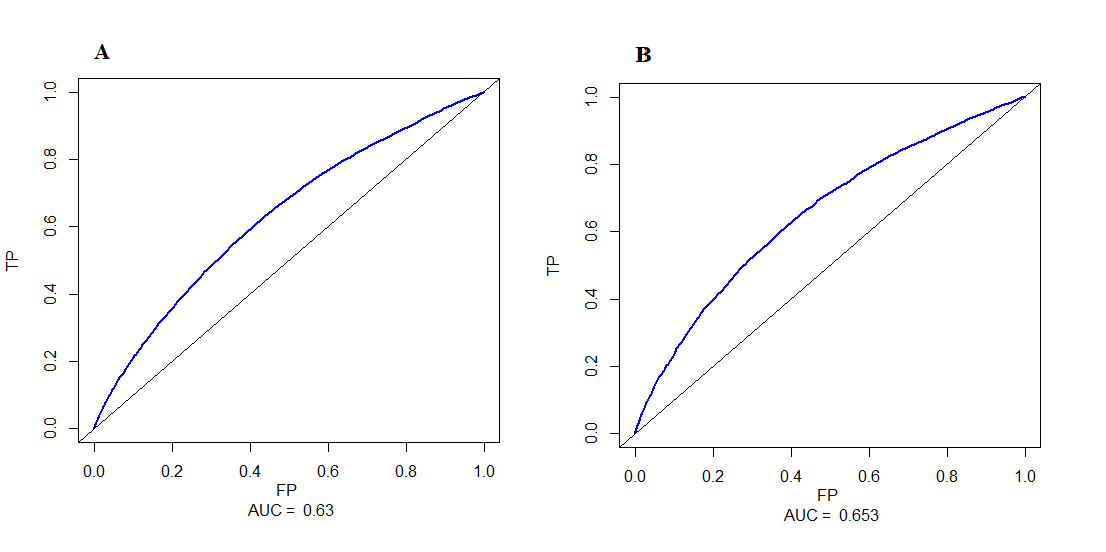

Supplement: Supplementary Figure 1 — The ROC curve in all EC patients. (A) The ROC curve for ACD. (B) The ROC curve for ESD. [file Image_1.tiff]
