# Supplementary material for: Association of Tumor Size With Prognosis in Patients With Resectable Endometrial Cancer: A SEER Database Analysis
Source: Front Oncol. 2022 Jun 23;12:887157. doi: 10.3389/fonc.2022.887157 (PMC9259839; doi:10.3389/fonc.2022.887157)
Supplement: Supplementary file 2 [file Table_1.docx]

**Supplementary Table 1. Multivariate Cox regression analyses for tumor size as continuous variable in all patients.**

| All-Cause Death | | | Endometrial Cancer-Specific Death | | |
| --- | --- | --- | --- | --- | --- |
| Factor | HR (95% CI) | *P* | Factor | HR (95% CI) | *P* |
| **Age** | 2.136 (2.079-2.195) | < 0.05 | **Age** | 1.558 (1.504-1.613) | <0.05 |
| **Tumor size** | 1.061 (1.053-1.069) | < 0.05 | **Tumor size** | 1.062 (1.052-1.073) | <0.05 |
| **Nodes examined** | 0.870 (0.847-0.893) | < 0.05 | **Nodes examined** | 0.898 (0.869-0.928) | <0.05 |
| **Race** |  | < 0.05 | **Race** |  | <0.05 |
| White | 1 |  | White | 1 |  |
| Black | 1.402 (1.321-1.488) |  | Black | 1.405 (1.307-1.511) |  |
| Others | 0.949 (0.885-1.019) | 0.151 | Others | 0.975 (0.894-1.064) | 0.569 |
| **Grade** |  | <0.05 | **Grade** |  |  |
| G1 | 1 |  | G1 | 1 | <0.05 |
| G2 | 1.343 (1.268-1.423) |  | G2 | 1.784 (1.626-1.956) |  |
| G3 | 2.250 (2.126-2.382) |  | G3 | 3.887 (3.557-4.246) |  |
| G4 | 2.501 (2.327-2.686) |  | G4 | 4.317 (3.890-4.790) |  |
| **Stage** |  | <0.05 | **Stage** |  | <0.05 |
| Ⅰ | 1 |  | Ⅰ | 1 |  |
| Ⅱ | 1.542 (1.442-1.649) |  | Ⅱ | 1.999 (1.823-2.192) |  |
| Ⅲ | 2.006 (1.887-2.133) |  | Ⅲ | 3.025 (2.799-3.269) |  |
| Ⅳ | 5.316 (4.930-5.732) |  | Ⅳ | 8.313 (7.601-9.092) |  |
| **Lymph node status** |  | <0.05 | **Lymph node status** |  | <0.05 |
| Negative | 1 |  | Negative | 1 |  |
| Positive | 1.521 (1.431-1.617) |  | Positive | 1.561 (1.456-1.674) |  |
| **Tumor number** |  | <0.05 | **Histological type** |  | <0.05 |
| Single | 1 |  | EEA | 1 |  |
| Multiple | 1.327 (1.272-1.383) |  | SEA | 1.115 (1.045-1.191) |  |

EEA, endometrial endometrioid adenocarcinoma; SEA, serous endometrioid adenocarcinoma.

**Supplementary Table 2. Multivariate Cox regression analyses for tumor size as categorical variable in all patients**

| All-Cause Death | | | Endometrial Cancer-Specific Death | | |
| --- | --- | --- | --- | --- | --- |
| Factor | HR (95%CI) | *P* | Factor | HR (95%CI) | *P* |
| **Age** | 2.152 (2.093-2.212) | <0.05 | **Age** | 1.574 (1.520-1.630) | <0.05 |
| **Nodes examined** | 0.870 (0.847-0.893) | <0.05 | **Nodes examined** | 0.901 (0.872-0.932) | <0.05 |
| **Race** |  | <0.05 | **Race** |  | <0.05 |
| White | 1 |  | White | 1 |  |
| Black | 1.347 (1.269-1.431) | <0.05 | Black | 1.351 (1.256-1.454) |  |
| Others | 0.946 (0.881-1.015) | 0.121 | Others | 0.970 (0.889-1.058) | 0.496 |
| **Grade** |  | <0.05 | **Grade** |  |  |
| G1 | 1 |  | G1 | 1 |  |
| G2 | 1.322 (1.248-1.400) |  | G2 | 1.760 (1.605-1.931) |  |
| G3 | 2.164 (2.042-2.292) |  | G3 | 3.781 (3.460-4.131) |  |
| G4 | 2.364 (2.192-2.550) |  | G4 | 4.175 (3.762-4.634) |  |
| **Stage** |  | <0.05 | **Stage** |  | <0.05 |
| Ⅰ | 1 |  | Ⅰ | 1 |  |
| Ⅱ | 1.452 (1.358-1.554) |  | Ⅱ | 1.877 (1.710-2.060) |  |
| Ⅲ | 1.892 (1.779-2.013) |  | Ⅲ | 2.841 (2.627-3.072) |  |
| Ⅳ | 4.855 (4.496-5.242) |  | Ⅳ | 7.603 (6.942-8.327) |  |
| **Tumor size group** |  | <0.05 | **Tumor size group** |  | <0.05 |
| ≤ 1 cm | 0.731 (0.659-0.811) |  | ≤ 1 cm | 0.744 (0.642-0.861) | 0.156 |
| 1.1-2 cm | 0.826 (0.767-0.891) |  | 1.1-2 cm | 0.829 (0.747-0.921) |  |
| 2.1-3 cm | 0.880 (0.824-0.939) |  | 2.1-3 cm | 0.864 (0.789-0.946) |  |
| 3.1-4 cm | 1 (reference) |  | 3.1-4 cm | 1 (reference) |  |
| 4.1-5 cm | 1.151 (1.080-1.227) |  | 4.1-5 cm | 1.170 (1.075-1.273) |  |
| 5.1-6 cm | 1.230 (1.145-1.321) |  | 5.1-6 cm | 1.232 (1.123-1.352) |  |
| 6.1-7 cm | 1.335 (1.230-1.449) |  | 6.1-7 cm | 1.406 (1.270-1.557) |  |
| 7.1-8 cm | 1.317 (1.196-1.450) |  | 7.1-8 cm | 1.378 (1.226-1.549) |  |
| 8.1-9 cm | 1.416 (1.262-1.589) |  | 8.1-9 cm | 1.514 (1.323-1.733) |  |
| > 9 cm | 1.613 (1.478-1.760) |  | > 9 cm | 1.614 (1.455-1.790) |  |
| **Lymph node status** |  | <0.05 | **Lymph node status** |  | <0.05 |
| Negative | 1 |  | Negative | 1 |  |
| Positive | 1.464 (1.377-1.556) |  | Positive | 1.503 (1.402-1.612) |  |
| **Histological type** |  | <0.05 | **Histological type** |  | <0.05 |
| EEA | 1 |  | EEA | 1 |  |
| SEA | 1.088 (1.028-1.151) |  | SEA | 1.159 (1.086-1.238) |  |
| **Tumor number** |  |  |  |  |  |
| Single | 1 |  |  |  |  |
| Multiple | 1.333 (1.278-1.390) |  |  |  |  |

EEA, endometrial endometrioid adenocarcinoma; SEA, serous endometrioid adenocarcinoma.

**Supplementary Table 3 Multivariate Cox regression analyses for tumor size as continuous variable in LNM**

| All-Cause Death | | | Endometrial Cancer-Specific Death | | |
| --- | --- | --- | --- | --- | --- |
| Factor | HR (95%CI) | *P* | Factor | HR (95%CI) | *P* |
| **Age** | 1.401 (1.326-1.481) |  | **Age** | 1.405 (1.330-1.484) | <0.05 |
| **Tumor size** | 1.047 (1.032-1.062) |  | **Tumor size** | 1.047 (1.032-1.063) | <0.05 |
| **Nodes examined** | 0.889 (0.846-0.934) |  | **Nodes examined** | 0.882 (0.839-0.926) | <0.05 |
| **Histological type** |  | <0.05 | **Histological type** |  | <0.05 |
| EEA | 1 |  | EEA | 1 |  |
| SEA | 1.129 (1.029-1.238) |  | SEA | 1.143 (1.042-1.253) |  |
| **Grade** |  | <0.05 | **Grade** |  | <0.05 |
| G1 | 1 |  | G1 | 1 |  |
| G2 | 1.523 (1.262-1.838) |  | G2 | 1.544 (1.279-1.863) |  |
| G3 | 3.068 (2.575-3.657) |  | G3 | 3.171 (2.662-3.778) |  |
| G4 | 3.369 (2.782-4.079) | 0.1181 | G4 | 3.484 (2.879-4.217) |  |
|  |  |  | **Tumor number** |  | <0.05 |
|  |  |  | Single | 1 |  |
|  |  |  | Multiple | 0.776 (0.701-0.859) |  |

EEA, endometrial endometrioid adenocarcinoma; SEA, serous endometrioid adenocarcinoma.

**Supplementary Table 4. Multivariate Cox regression analyses for tumor size as categorical variable in LNM**

| All-Cause Death | | | Endometrial Cancer-Specific Death | | |
| --- | --- | --- | --- | --- | --- |
| Factor | HR (95%CI) | *P* | Factor | HR (95%CI) | *P* |
| **Age** | 1.605 (1.527-1.687) | <0.05 | **Age** | 1.438 (1.361-1.519) | <0.05 |
| **Nodes examined** | 0.879 (0.840-0.920) | <0.05 | **Nodes examined** | 0.892 (0.849-0.937) | <0.05 |
| **Grade** |  | <0.05 | **Grade** |  | <0.05 |
| G1 | 1 |  | G1 | 1 |  |
| G2 | 1.309 (1.124-1.525) |  | G2 | 1.555 (1.289-1.876) |  |
| G3 | 2.359 (2.048-2.719) |  | G3 | 3.112 (2.612-3.707) |  |
| G4 | 2.561 (2.185-3.001) |  | G4 | 3.380 (2.792-4.092) |  |
| **Tumor size group** |  |  | **Tumor size group** |  | <0.05 |
| ≤ 1 cm | 1.254 (0.982-1.601) | 0.336 | ≤ 1 cm | 1.156 (0.953-1.401) | 0.059 |
| 1.1-2 cm | 1.097 (0.922-1.306) | 0.087 | 1.1-2 cm | 0.978 (0.830-1.153) |  |
| 2.1-3 cm | 1.004 (0.869-1.161) | 0.070 | 2.1-3 cm | 1.108 (0.962-1.275) |  |
| 3.1-4 cm | 1 (reference) |  | 3.1-4 cm | 1 (reference) |  |
| 4.1-5 cm | 1.112 (0.981-1.259) | 0.331 | 4.1-5 cm | 1.195 (1.031-1.385) |  |
| 5.1-6 cm | 1.175 (1.029-1.341) | 0.604 | 5.1-6 cm | 1.360 (1.166-1.586) |  |
| 6.1-7 cm | 1.320 (1.149-1.516) | 0.687 | 6.1-7 cm | 1.304 (1.094-1.554) |  |
| 7.1-8 cm | 1.279 (1.092-1.499) | 0.880 | 7.1-8 cm | 1.395 (1.150-1.693) |  |
| 8.1-9 cm | 1.359 (1.138-1.624) | 0.562 | 8.1-9 cm | 1.702 (1.471-1.970) |  |
| > 9 cm | 1.286 (0.983-1.681) |  | > 9 cm | 1.156 (0.953-1.401) |  |
| **Histological type** |  | <0.05 | **Tumor number** |  | <0.05 |
| EEA | 1 |  | Single | 1 |  |
| SEA | 1.120 (1.028-1.220) |  | Multiple | 0.776 (0.701-0.859) |  |
|  |  |  | **Histological type** |  | <0.05 |
|  |  |  | EEA | 1 |  |
|  |  |  | SEA | 1.171 (1.067-1.285) |  |

EEA, endometrial endometrioid adenocarcinoma; SEA, serous endometrioid adenocarcinoma.

**Supplementary Table 5. Multivariate Cox regression analyses for tumor size as continuous variable in non-LNM**

| All-Cause Death | | | Endometrial Cancer-Specific Death | | |
| --- | --- | --- | --- | --- | --- |
| Factor | HR (95%CI) | *P* | Factor | HR (95%CI) | *P* |
| **Age** | 2.413 (2.335-2.494) | <0.05 | **Age** | 1.669 (1.595-1.747) | <0.05 |
| **Tumor size** | 1.067 (1.057-1.077) | <0.05 | **Tumor size** | 1.075 (1.061-1.088) | <0.05 |
| **Nodes examined** | 0.874 (0.846-0.903) | <0.05 | **Nodes examined** | 0.909 (0.869-0.950) | <0.05 |
| **Histological type** |  | <0.05 | **Histological type** |  | <0.05 |
| EEA | 1 |  | EEA | 1 |  |
| SEA | 1.486 (1.416-1.559) |  | SEA | 1.126 (1.026-1.235) |  |
| **Tumor number** |  | <0.05 | **Tumor number** |  | <0.05 |
| Single |  |  | Single | 1 |  |
| Multiple | 1.486 (1.416-1.559) |  | Multiple | 1.121 (1.042-1.205) |  |
| **Race** |  | <0.05 | **Race** |  | <0.05 |
| White |  |  | White | 1 |  |
| Black | 1.456 (1.352-1.569) |  | Black | 1.488 (1.349-1.642) |  |
| Others | 0.993 (0.911-1.083) | 0.871 | Others | 1.049 (0.935-1.177) | 0.418 |
| **Grade** |  | <0.05 | **Grade** |  |  |
| G1 |  |  | G1 |  |  |
| G2 | 1.345 (1.264-1.432) |  | G2 | 1.818 (1.634-2.022) | <0.05 |
| G3 | 2.152 (2.017-2.296) |  | G3 | 4.121 (3.717-4.568) |  |
| G4 | 2.351 (2.145-2.577) |  | G4 | 4.614 (4.055-5.250) |  |
| **Stage** |  | <0.05 | **Stage** |  | <0.05 |
| Ⅰ |  |  | Ⅰ | 1 |  |
| Ⅱ | 1.541 (1.441-1.648) |  | Ⅱ | 1.967 (1.793-2.158) |  |
| Ⅲ | 2.117 (1.981-2.262) |  | Ⅲ | 2.998 (2.753-3.264) |  |
| Ⅳ | 5.202 (4.715-5.740) |  | Ⅳ | 8.336 (7.461-9.313) |  |

EEA, endometrial endometrioid adenocarcinoma; SEA, serous endometrioid adenocarcinoma.

**Supplementary Table 6. Multivariate Cox regression analyses for tumor size as categorical variable in non-LNM**

| All-Cause Death | | | Endometrial Cancer-Specific Death | | |
| --- | --- | --- | --- | --- | --- |
| Factor | HR (95% CI) | *P* | Factor | HR (95% CI) | *P* |
| **Age** | 2.428 (2.349-2.509) | < 0.05 | **Age** | 1.679 (1.605-1.757) | <0.05 |
| **Nodes examined** | 0.870 (0.842-0.899) | <0.05 | **Nodes examined** | 0.906 (0.867-0.948) | <0.05 |
| **Race** |  | <0.05 | **Race** |  | <0.05 |
| White | 1 |  | White | 1 |  |
| Black | 1.389 (1.289-1.498) |  | Black | 1.406 (1.273-1.552) |  |
| Others | 0.998 (0.915-1.088) | 0.961 | Others | 1.049 (0.935-1.178) | 0.412 |
| **Grade** |  | <0.05 | **Grade** |  | <0.05 |
| G1 | 1 |  | G1 | 1 |  |
| G2 | 1.304 (1.225-1.388) |  | G2 | 1.760 (1.582-1.959) |  |
| G3 | 2.064 (1.934-2.202) |  | G3 | 3.951 (3.563-4.381) |  |
| G4 | 2.265 (2.066-2.483) |  | G4 | 4.457 (3.916-5.072) |  |
| **Stage** |  | <0.05 | **Stage** |  | <0.05 |
| Ⅰ | 1 |  | Ⅰ | 1 |  |
| Ⅱ | 1.442 (1.348-1.543) |  | Ⅱ | 1.823 (1.660-2.002) |  |
| Ⅲ | 1.982 (1.854-2.120) |  | Ⅲ | 2.782 (2.552-3.032) |  |
| Ⅳ | 4.783 (4.329-5.283) |  | Ⅳ | 7.528 (6.725-8.427) |  |
| **Tumor size group** |  | <0.05 | **Tumor size group** |  |  |
| ≤ 1 cm | 0.658 (0.587-0.738) |  | ≤ 1 cm | 0.732 (0.646-0.829) | 0.066 |
| 1.1-2 cm | 0.771 (0.710-0.838) |  | 1.1-2 cm | 0.811 (0.727-0.905) |  |
| 2.1-3 cm | 0.840 (0.781-0.904) |  | 2.1-3 cm | 1.211 (1.090-1.347) |  |
| 3.1-4 cm | 1 (reference) |  | 3.1-4 cm | 1 (reference) |  |
| 4.1-5 cm | 1.175 (1.090-1.266) |  | 4.1-5 cm | 1.267 (1.123-1.429) |  |
| 5.1-6 cm | 1.262 (1.159-1.375) |  | 5.1-6 cm | 1.468 (1.278-1.685) |  |
| 6.1-7 cm | 1.382 (1.246-1.533) |  | 6.1-7 cm | 1.491 (1.273-1.747) |  |
| 7.1-8 cm | 1.350 (1.192-1.528) |  | 7.1-8 cm | 1.680 (1.386-2.037) |  |
| 8.1-9 cm | 1.454 (1.245-1.698) |  | 8.1-9 cm | 1.443 (1.228-1.696) |  |
| > 9 cm | 1.457 (1.284-1.653) |  | > 9 cm | 1.702 (1.471-1.970) |  |
| **Tumor number** |  | <0.05 | **Tumor number** |  | <0.05 |
| Single | 1 |  | Single | 1 |  |
| Multiple | 1.501 (1.430-1.575) |  | Multiple | 1.131 (1.052-1.216) |  |
| **Histological type** |  | <0.05 | **Histological type** |  | <0.05 |
| EEA | 1 |  | EEA | 1 |  |
| SEA | 1.097 (1.016-1.184) |  | SEA | 1.190 (1.084-1.306) |  |

EEA, endometrial endometrioid adenocarcinoma; SEA, serous endometrioid adenocarcinoma.
